# Population-based estimates of the prevalence of *FMR1* expansion mutations in women with early menopause and primary ovarian insufficiency

**DOI:** 10.1038/gim.2013.64

**Published:** 2013-05-23

**Authors:** Anna Murray, Minouk J. Schoemaker, Claire E. Bennett, Sarah Ennis, James N. Macpherson, Michael Jones, Danielle H. Morris, Nick Orr, Alan Ashworth, Patricia A. Jacobs, Anthony J. Swerdlow

**Affiliations:** 1 University of Exeter Medical School, University of Exeter St Luke's, Exeter, UK; 2 Division of Genetics and Epidemiology, The Institute of Cancer Research, Sutton, UK; 3 Genetic Epidemiology, University of Southampton, Southampton, UK; 4 Wessex Regional Genetics Laboratory, Salisbury District Hospital, Salisbury, UK; 5 Division of Breast Cancer Research, The Institute of Cancer Research, London, UK

**Keywords:** expansion, *FMR1*, menopause, POI, premutation

## Abstract

**Purpose:**

Primary ovarian insufficiency before the age of 40 years affects 1% of the female population and is characterized by permanent cessation of menstruation. Genetic causes include *FMR1* expansion mutations. Previous studies have estimated mutation prevalence in clinical referrals for primary ovarian insufficiency, but these are likely to be biased as compared with cases in the general population. The prevalence of *FMR1* expansion mutations in early menopause (between the ages of 40 and 45 years) has not been published.

**Methods:**

We studied *FMR1* CGG repeat number in more than 2,000 women from the Breakthrough Generations Study who underwent menopause before the age of 46 years. We determined the prevalence of premutation (55–200 CGG repeats) and intermediate (45–54 CGG repeats) alleles in women with primary ovarian insufficiency (*n* = 254) and early menopause (*n* = 1,881).

**Results:**

The prevalence of the premutation was 2.0% in primary ovarian insufficiency, 0.7% in early menopause, and 0.4% in controls, corresponding to odds ratios of 5.4 (95% confidence interval = 1.7–17.4; *P* = 0.004) for primary ovarian insufficiency and 2.0 (95% confidence interval = 0.8–5.1; *P* = 0.12) for early menopause. Combining primary ovarian insufficiency and early menopause gave an odds ratio of 2.4 (95% confidence interval = 1.02–5.8; *P* = 0.04). Intermediate alleles were not significant risk factors for either early menopause or primary ovarian insufficiency.

**Conclusion:**

*FMR1* premutations are not as prevalent in women with ovarian insufficiency as previous estimates have suggested, but they still represent a substantial cause of primary ovarian insufficiency and early menopause.

The polymorphic CGG trinucleotide repeat in the *FMR1* gene is associated with three different phenotypes.^1^ Full mutations have over 200 CGG repeats and are usually fully methylated, causing inactivation of the gene. Loss-of-function mutations in *FMR1* give rise to fragile X syndrome, characterized by moderate-to-severe learning difficulties and social deficits and more often affecting males than females. A premutation category of smaller repeats, with 55–200 CGGs, is associated with fragile X tremor and ataxia syndrome and primary ovarian insufficiency (POI). Fragile X tremor and ataxia syndrome is a late-onset progressive neurological disorder that affects mainly males.^2^ POI is defined as permanent cessation of menstruation occurring naturally before the age of 40 years.^3^ POI affects ~1% of the general female population but has been reported in ~24% of premutation carriers in fragile X families.^4,5^ Women carrying premutation-sized repeats go through menopause ~5 years earlier than noncarriers in their families.^6^ Although the exact molecular mechanism by which *FMR1* premutations affect phenotype is unknown, there is substantial evidence for an RNA gain-of-function effect, as only premutation and not full-mutation carriers are at risk for fragile X tremor and ataxia syndrome and POI, and RNA levels are increased in premutation carriers, whereas no RNA is produced from methylated full-mutation alleles.^5,7^

Several studies have investigated the prevalence of the *FMR1* premutation in series of women ascertained via POI. Other genetic causes of POI have been reported, including X chromosome abnormalities and mutations in genes such as *FOXL2* and *FSHR*. These represent <5% of cases, but in the majority of cases the cause is unknown.^8^ *FMR1* premutations are thought to account for ~5% of all POI cases and more if it is a familial condition.^6,9,10^ Several recent reports have also suggested that smaller repeats in the higher end of the normal range (intermediate or gray-zone alleles) also affect ovarian function.^11,12,13^ However, all previous studies on the incidence of premutation and intermediate alleles in POI have been in women who were having clinical investigations either for POI or infertility. These cases are selected and hence could have increased prior odds of finding a premutation, perhaps because of an undisclosed family history. Therefore, in the absence of a population-based estimate, it is difficult to assess the impact of this mutation on reproductive life span. Moreover, the majority of previous studies have solely investigated women with POI, which has been fairly arbitrarily defined on the basis of the population distribution of menopause. We do not know whether women with early menopause (EM) after 40 years of age are also at significant risk of carrying a premutation. We therefore tested a series of women with menopause at or before 45 years of age from the population-based Breakthrough Generations Study (BGS) to determine the prevalence of *FMR1* CGG repeat expansion mutations, both in those having POI and in those having EM.

## Materials and Methods

### Study population

The BGS is a UK prospective epidemiological cohort study that started recruitment in 2003. The primary objective of the BGS is to investigate the environmental, behavioral, hormonal, and genetic causes of breast cancer and also investigate the causes of other cancers and diseases.^14^ The cohort consists of more than 110,000 women from the general population of the United Kingdom aged 16 years and older at the date of entry. Recruitment is through volunteers connected with the charity Breakthrough Breast Cancer, volunteers responding to publicity, and their friends, family members, and other contacts. Each participant completes a questionnaire, and most provide a blood sample for the analysis of genomic, hormonal, and other blood factors. Participants are questioned on their detailed menstrual histories, thus enabling the identification of the subjects in the current analyses. Natural menopause was defined as the cessation of menstruation for at least 6 months without known cause. Women were excluded if their periods stopped because of pregnancy, breastfeeding, surgery, hormonal contraceptive use, or other types of medical treatment, or if there was a medical condition or illness that could have caused amenorrhea (e.g., polycystic ovary syndrome). We also excluded women with a history of breast cancer as cases and controls. EM cases were women who had natural menopause between 40 and 45 years of age and POI cases had menopause before 40 years of age. We selected one control for each EM and POI case, at random within matching criteria of date of birth (within 12 months if possible), ethnicity, year of questionnaire completion, and source of recruitment. Overall, there were 126 cases who were younger than 46 years at entry to the study, and controls aged 46 years at entry to the study were selected for each of these cases. Women were eligible to be enrolled as controls if they were postmenopausal at entry to the study with a menopausal age of 46 years or older (74.3%) or if they were premenopausal and entered the study at the age of 46 years or older (25.7%). Menopause at the age of 46 years or older could be natural or surgically induced, provided there was evidence that the women were still menstruating after the age of 45 years. When multiple individuals from one pedigree were available, we included only the youngest individual who met the above criteria. We successfully genotyped 2,135 women with natural menopause before the age of 46 years and 1,915 controls with menopause after the age of 45 years; details are given in **Table 1**.

### Genotyping

For each subject, Asuragen Amplidex kits (http://www.asuragen.com/) containing *FMR1* CGG repeat region–specific primers were used to PCR amplify the *FMR1* repeat region from 20 ng of genomic DNA that had been extracted from peripheral blood mononuclear cells. All PCRs were performed in 3 µl of reaction volumes in 384-well microtiter plates, using conditions recommended by the kit manufacturers. Products were size separated by capillary electrophoresis on an ABI 3730 automated sequencer (Applied Biosystems, Warrington, UK), using ROX 1000 size standard (Asuragen, Austin, TX, USA) for estimation of product sizes. CGG repeat numbers were determined by comparison with a control individual of 52 CGG repeats. We included duplicates of 776 of the samples (20%) on independent plates. The concordance between duplicate samples was 98.5%, excluding differences of ±1 CGG repeat. Controls included 12 no-template controls, 3 samples from females of known expansion size (largest CGG = 55, 117, and 145), and a lane containing the multiple size targets supplied by Asuragen (CGG = 20, 29, 31, 53, 117, and 196) per 384 plate.

### Analysis methods and models

The association between CGG repeat length and age at natural menopause was tested by logistic and linear regression analysis in STATAv12 (http://www.stata.com). Models testing the CGG repeat as continuous and categorical variables were investigated against age at menopause as both continuous and categorical variables. We tested the effect of the longest CGG repeat allele on EM and POI, both with and without the smaller allele as a continuous variable covariate, the hypotheses being that expansions may act as dominant alleles or may act additively with the second, smaller repeat. The largest CGG repeat was categorized as premutation (55–200 CGG repeats) or intermediate (45–54 CGG repeats) based on accepted definitions (http://www.acog.org/), although these definitions are largely based on the thresholds that determine instability of the repeat and may not be as relevant for determining thresholds for phenotypic effects. For analysis, risk in relation to premutations was compared with subjects with <55 CGG repeats (i.e., including the intermediate category), and risk in relation to intermediate repeats (45–54) was compared with subjects with <45 CGG repeats.

Age at natural menopause was analyzed in two different ways:

1. As a quantitative trait using age at last menstrual period, in both a linear and quadratic model.
2. As a case–control outcome, with EM (40–46 years of age) as the cases, and then with POI (menopause before 40 years of age) as the cases. In both instances, women with menopause after 45 years constituted the control group. We also tested the frequency of CGG repeat expansions in EM and POI cases combined.

## Results

### Prevalence of *FMR1* expansion alleles in women with EM and POI

There was a significant excess of *FMR1* premutation-sized repeats in women with POI, with 2% having premutation-sized repeats as compared with 0.4% of controls (**Table 1**) (*P* = 0.008 by Fisher's exact test). In 0.7% of women with EM, we detected a *FMR1* premutation (*P* = 0.13 by Fisher's exact test). Women with POI had more than a fivefold increased odds of being premutation carriers (odds ratio (OR) = 5.4; 95% confidence interval (CI) = 1.7–17.4; *P* = 0.004), whereas women in the EM group were at twofold increased odds (OR = 2.0; 95% CI = 0.8–5.1; *P* = 0.12). When women with EM and POI were combined, i.e., for women with menopause occurring at <46 years, the odds of being a premutation carrier was >2 (OR = 2.4; 95% CI = 1.02–5.8; *P* = 0.04). Intermediate alleles were not significantly associated with either EM or POI (**Table 2**). The ORs were not substantially affected by including the smaller CGG repeat allele as a covariate (**Table 2**). There was no evidence for a continuous per-repeat effect of CGG number on menopause age or case/control status (data not shown). There was no linear or curvilinear association between age of menopause and number of CGG repeats in the premutation range (*P* = 0.29 and *P* = 0.28, respectively), but inclusion of intermediate alleles gave a better fit for the curvilinear model (*P* = 0.035).

Smoking is the most robustly associated environmental factor affecting menopause age,^15^ and the OR for being an EM or POI case for individuals who had ever smoked was OR = 1.3 (95% CI = 1.1–1.4; *P* = 0.0002). We investigated the potential impact of smoking status in a sensitivity analysis, repeating the association tests in women who had never smoked and women who had ever smoked over their lifetime in separate analyses: no significant differences were observed (**Supplementary Table S1** online). In an interaction analysis, a log likelihood ratio test found no evidence that the OR for POI or EM for premutation alleles was modified by smoking status (*P* = 0.29 and 0.71, respectively).

### Repeat sizes observed

**Figure 1:**
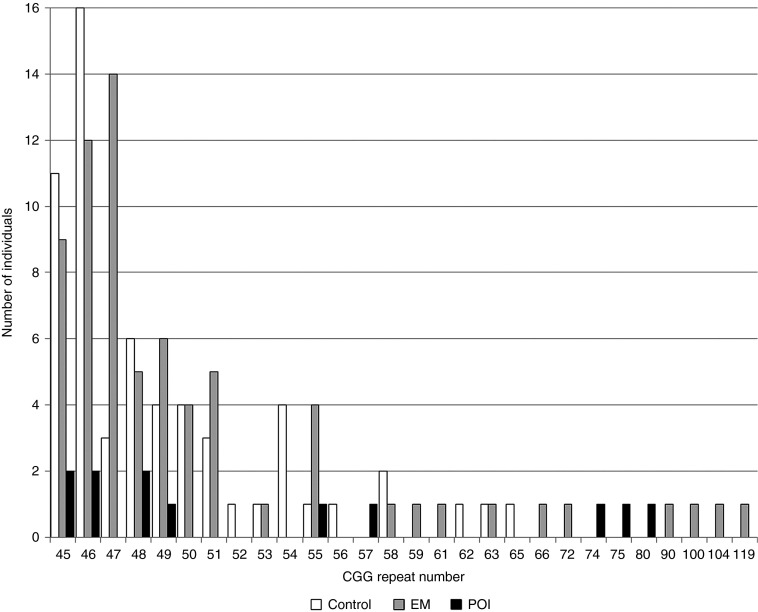
***FMR1* CGG repeat sizes >45, for the largest allele, in women with POI, EM, and menopause after 45 years of age (controls).** EM, early menopause; POI, primary ovarian insufficiency.

The expanded premutation-sized alleles observed in controls were all <66 repeats. The larger premutation alleles (>65 repeats) were all in women with either EM or POI (**Figure 1**).

## Discussion

We found an excess of *FMR1* premutation alleles in both women with EM and women with POI in our large sample of individuals. Although the BGS cohort consists of volunteers from, rather than random members of, the general population, they included substantial numbers from all sections of society and geographical areas of the UK, and it is highly unlikely that participants volunteered on the basis of the combination of their menopausal status and *FMR1* status (which would be unknown to them), so they probably give a close estimate of the association in the population overall. A limitation of this study is that it relied on recalled menopause age. Recall of menopause age has been reported to be reliable in other studies,^16^ but recall errors could lead to misclassification when the data were grouped as POI, EM, or controls. If such misclassification occurred nondifferentially, then it would be expected to reduce the ORs.

Previous estimates of the frequency of premutation alleles in women ascertained through POI range from 0 to 10%, with a combined prevalence across published studies for 55–200 repeat alleles of ~4% (**Supplementary Table S2** online). The prevalence is reported as being higher in individuals with a family history of POI, although most studies did not report data for familial and sporadic cases separately. To our knowledge, this is the first population-based study that examined individuals at the extreme end of the general population menopause age distribution rather than clinical referrals for EM. We found a fivefold increased odds of having POI in female carriers of a premutation allele as compared with a 10-fold increased odds in our previous studies in an independent group of patients ascertained through clinical referral for POI, but the 95% CI in our current study includes an OR of 10.^10,17^ It is possible that clinical referrals are enriched for cases at the severe end of the menopause spectrum, or with a family history, and thus may be more likely to have a genetic etiology. Differences between studies may reflect true phenotypic differences in the women classified as POI. Some studies report families that included fragile X–affected males^13,18,19^ who were diagnosed before testing females with POI and thus it is difficult to be certain that the index POI case would have been ascertained independently; these cases may therefore increase the prior odds of finding a premutation.^18^ Alternatively, there may be a reporting bias, whereby studies with negative or borderline associations are less likely to be published. A population-based sample, unbiased with regard to menopause or fragile X status, should therefore give the best estimate of the prevalence of the expansion mutation.

**Figure 2:**
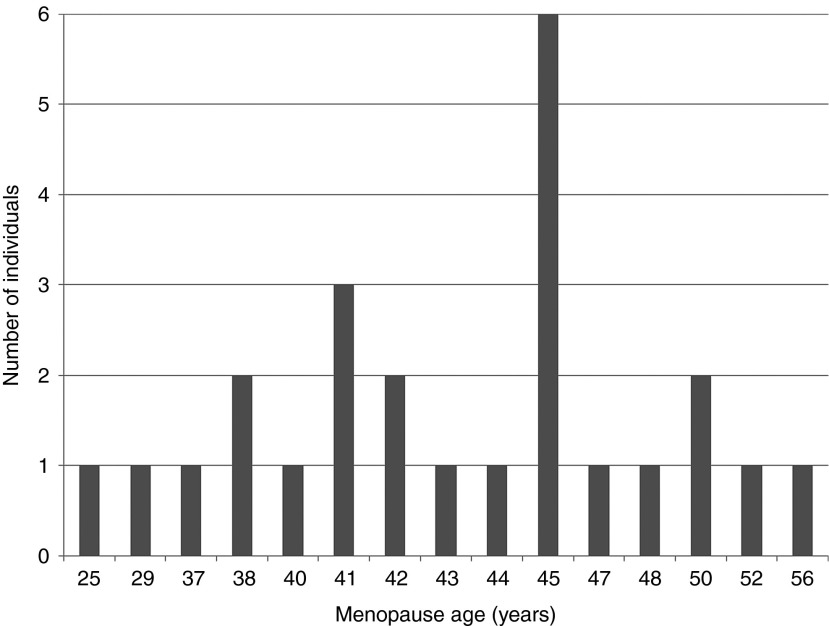
**Menopause age in premutation carriers (*n* = 25).**

This is the first study that has tested a large number of women with EM as well as those with POI. These women might not seek medical attention but represent 10% of the women recruited at ages of 60 years or more in our BGS population. The raised OR in premutation carriers in our study was not statistically significant, but if confirmed in future studies, then a much larger number of individuals may be affected by premutations than would be the case if the association were limited to women with POI. For three of the women with EM in our data, the premutation allele was 100 repeats or greater, a size that in families identified through fragile X syndrome would almost always expand to a full mutation during a single maternal transmission. Other alleles were at the lower end of the premutation spectrum, with fewer than 60 CGG repeats. We were not able to follow up these alleles in family-based studies, but it is important to determine whether premutation alleles ascertained in this way, i.e., in the absence of a full mutation, are equally unstable. There is some evidence to suggest that these alleles may be more stable, but further studies are required.^20^ It is difficult to recommend testing for these mutations routinely until the implications of finding premutations in females with EM are fully understood. On the basis of prevalence alone, testing the *FMR1* gene in women with EM would have as good a detection rate for the premutation as screening children with developmental delay/learning difficulties has for the full mutation; therefore, many new families that segregate the mutation could be identified by testing women with EM. The counseling of families identified in this manner will be substantially different from the counseling of families with fragile X syndrome, and the uptake of family testing is also likely to be different. In many cases, the reason for testing will not be to provide reproductive advice to the index case with EM, as she will generally not be able to conceive naturally, but the diagnosis will have implications for the wider family, for both reproductive health and the possibility of having offspring with fragile X syndrome. Although premutation-sized *FMR1* repeats are substantial risk factors for EM and POI, premutations are also found in women with menopause occurring after 50 years of age (**Figure 2**), and therefore, it is possible that additional causative mutations are present in EM and POI cases, and further diagnostic testing should not be disregarded.

Previous studies have reported an increased frequency of intermediate-sized *FMR1* alleles in cases of POI and EM.^12,13^ This would fit with a molecular model whereby a linear increase in RNA levels with increasing CGG repeat number has a linear effect on menopause age. Previous studies from our own lab^21^ and the current study were not able to confirm an association between EM/POI and intermediate/large normal *FMR1* alleles. Together, these studies were considerably larger than other published reports, but it is unclear how the study designs differ. It is possible that there are differences in specific populations tested, given that both of our studies were in UK-based cohorts, whereas other reports have come from Italy, Canada, and the United States, or again there might be positive reporting bias. The largest study implicating intermediate alleles in ovarian function came from an American group that tested 535 women with occult POI, i.e., who were still cycling but had raised follicle-stimulating hormone or poor response to ovarian stimulation.^11^ The prevalence of intermediate alleles was 3.2% in these women as compared with 1.3% in controls (*P* = 0.046). In the current study of 1,881 EM cases and 254 POI cases, no excess of intermediate alleles was seen, and there was no evidence for a quantitative, per-repeat, effect on menopause age. Our study was sufficiently powered to detect an association with intermediate alleles, with >80% power to detect an OR of 1.5 for POI in intermediate carriers, whereas previous studies have reported ORs of 2.4–4.8.^11,12,13^ Therefore, although we cannot discount an association, we suggest the effect of intermediate alleles on ovarian function is at most weaker than previous studies would suggest. Two studies have reported a nonlinear effect of premutation size on menopause age, and therefore, a simple relationship between mRNA levels and menopause age may not explain the association with *FMR1* expansions.^22,23^ In this study, we found no correlation between CGG number and menopause age within the premutation category, but we did not have many cases of large premutation carriers and were not as well powered as previous studies to detect an association. We did however find a borderline significant association with CGG repeat number >44 in the curvilinear model.

In summary, *FMR1* premutations may not be as common a cause of ovarian failure as previous estimates suggest, but they still represent an important cause of ovarian failure and therefore give a reason to test women with POI and EM for the mutation.

## Disclosure

The authors declare no conflict of interests.

## Figures and Tables

**Table 1:**
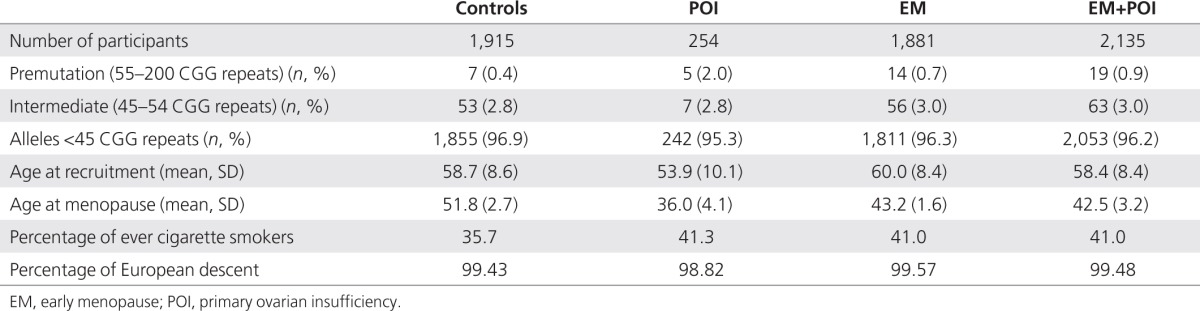
Summary statistics for individuals included in the analysis

**Table 2:**
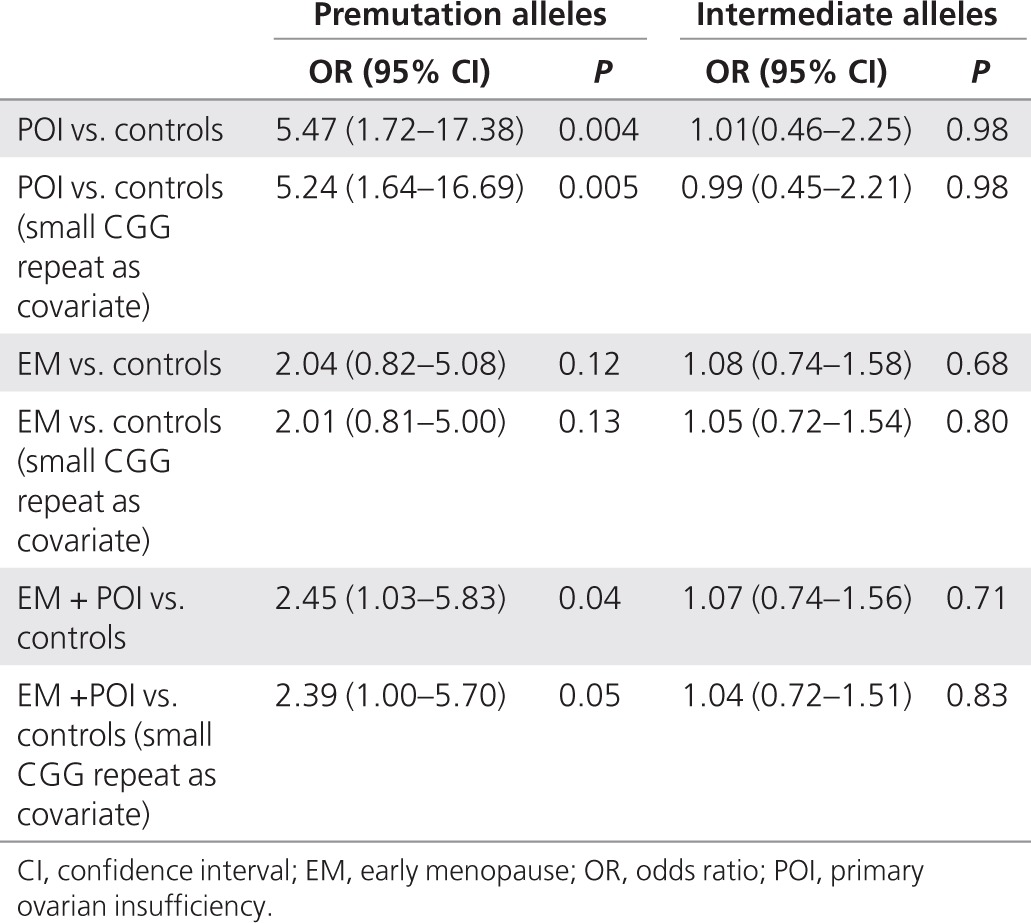
Odds ratio for EM or POI as compared with controls for carriers of premutation and intermediate *FMR1* alleles
